# Engineering of glycerol utilization in *Gluconobacter oxydans* 621H for biocatalyst preparation in a low-cost way

**DOI:** 10.1186/s12934-018-1001-0

**Published:** 2018-10-08

**Authors:** Jinxin Yan, Jing Xu, Menghao Cao, Zhong Li, Chengpeng Xu, Xinyu Wang, Chunyu Yang, Ping Xu, Chao Gao, Cuiqing Ma

**Affiliations:** 1 State Key Laboratory of Microbial Technology & Shenzhen Research Institute, Shandong University, 27 Shanda South Road, Jinan, 250100 People’s Republic of China; 2 State Key Laboratory of Microbial Metabolism, Joint International Research Laboratory of Metabolic & Developmental Sciences, and School of Life Sciences & Biotechnology, Shanghai Jiao Tong University, 800 Dongchuan Road, Shanghai, 200240 People’s Republic of China; 3 Dong Ying Oceanic and Fishery Bureau, 206 Yellow River Road, Dongying, 257091 People’s Republic of China

**Keywords:** *Gluconobacter oxydans* 621H, Glycerol, Biocatalysis, Xylonate

## Abstract

**Background:**

Whole cells of *Gluconobacter oxydans* are widely used in various biocatalytic processes. Sorbitol at high concentrations is commonly used in complex media to prepare biocatalysts. Exploiting an alternative process for preparation of biocatalysts with low cost substrates is of importance for industrial applications.

**Results:**

* G. oxydans* 621H was confirmed to have the ability to grow in mineral salts medium with glycerol, an inevitable waste generated from industry of biofuels, as the sole carbon source. Based on the glycerol utilization mechanism elucidated in this study, the major polyol dehydrogenase (GOX0854) and the membrane-bound alcohol dehydrogenase (GOX1068) can competitively utilize glycerol but play no obvious roles in the biocatalyst preparation. Thus, the genes related to these two enzymes were deleted. Whole cells of *G. oxydans* ∆GOX1068∆GOX0854 can be prepared from glycerol with a 2.4-fold higher biomass yield than that of *G. oxydans* 621H. Using whole cells of *G. oxydans* ∆GOX1068∆GOX0854 as the biocatalyst, 61.6 g L^−1^ xylonate was produced from 58.4 g L^−1^ xylose at a yield of 1.05 g g^−1^.

**Conclusion:**

This process is an example of efficient preparation of whole cells of *G. oxydans* with reduced cost. Besides xylonate production from xylose, other biocatalytic processes might also be developed using whole cells of metabolic engineered *G. oxydans* prepared from glycerol.

**Electronic supplementary material:**

The online version of this article (10.1186/s12934-018-1001-0) contains supplementary material, which is available to authorized users.

## Background

*Gluconobacter oxydans* is capable of incompletely oxidizing a large variety of carbohydrates and alcohols [1–3]. Whole cells of *G. oxydans* have been widely used in production of a variety of compounds such as vitamin C, dihydroxyacetone (DHA), xylonate and gluconate [4–8]. Traditionally, *G. oxydans* is cultivated in complex media with high concentrations of sorbitol and yeast extract [9]. The high biocatalyst manufacturing cost might hamper the scale-up application of *G. oxydans* in biotransformation. To cope with this problem, several studies were carried out to prepare whole cells of *G. oxydans* from other carbon sources. For example, the membrane-bound glucose dehydrogenase (GDHK) in *G. oxydans* 621H was disrupted, and the mutant strain was laboratory-evolved with glucose as the sole carbon source and yeast extract as the nitrogen source. Whole cells of the evolved strain grown on a low concentration of glucose exhibited similar catalytic efficiency with the wild-type strain cultured on high concentrations of sorbitol [10].

Glycerol is an inevitable waste generated during the production of biofuels such as bioethanol and biodiesel [11–15]. Approximately 1 kg of crude glycerol is produced during the production of 9 kg of biodiesel [16]. The global annual production of glycerol is estimated to reach approximately 4.2 million tons in 2020 (OECD/FAO 2012) [17]. The existing market for industrial application of glycerol is only 1 million tons/year [13]. Therefore, it is desirable to develop new processes that can convert glycerol into high-value products such as DHA and glycerate [18, 19]. Besides an attractive substrate for the production of value-added products [19–21], glycerol can also be used as a cheap carbon source to culture various microbes (Additional file 1: Table S1). Whole cells prepared from glycerol can be used in lipids extraction (oil-accumulating microbes) or biocatalysis processes (microbes with different enzymes) [22–25].

**Fig. 1:**
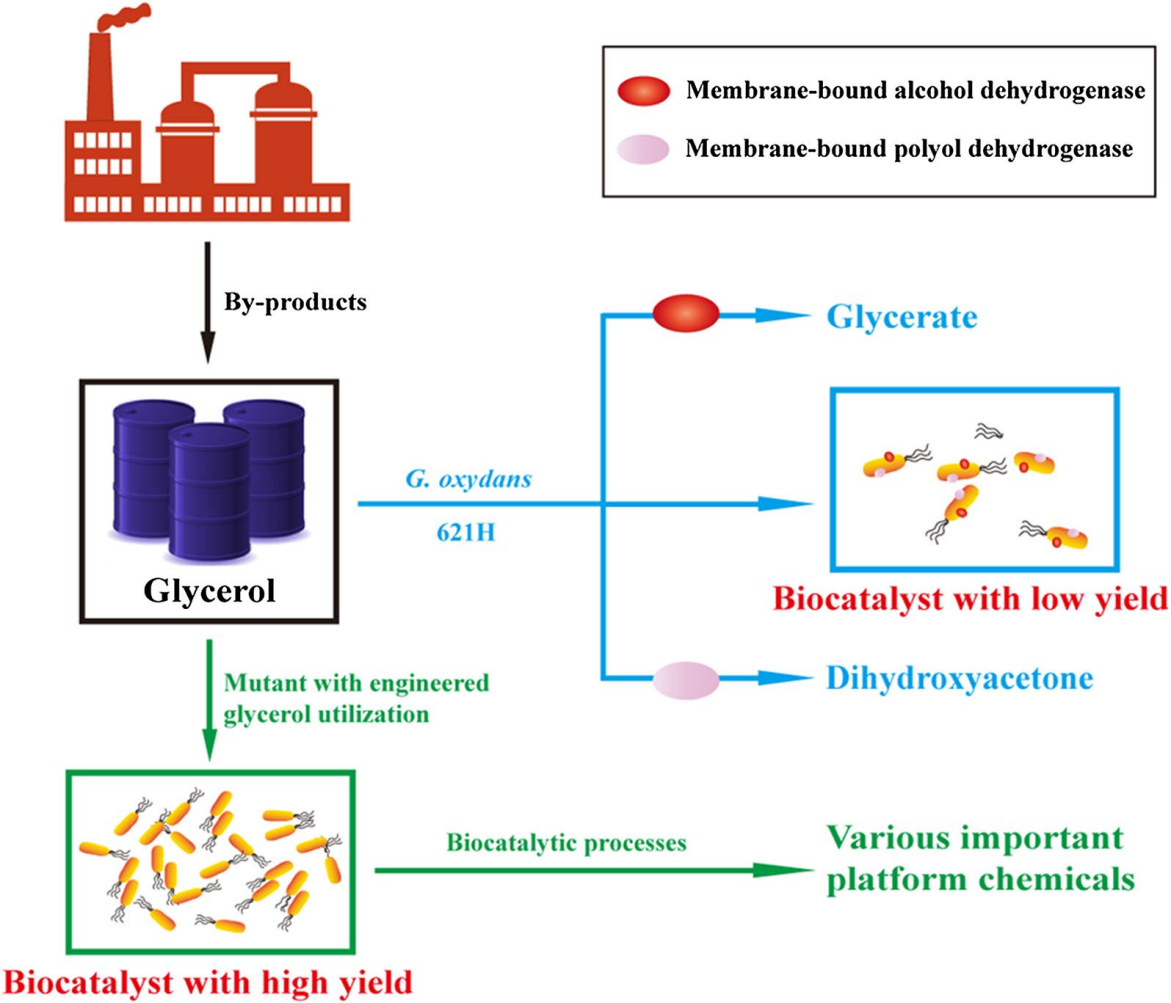
Scheme for the efficient preparation of whole-cell biocatalyst of *G. oxydans* 621H from biofuel-industry generated glycerol

* G. oxydans* 621H is able to utilize glycerol as the energy source in complex media and producing glycerate and DHA as the glycerol oxidation products [26–29]. Preparing whole cells of *G. oxydans* as biocatalysts using glycerol as the sole carbon source in mineral salts media is desirable for industrial processes. In this study, the glycerol utilization mechanism of *G. oxydans* 621H was elucidated. Then, a mutant strain that can grow with low glycerol consumption but high yield of biomass was constructed (Fig. 1). Whole cells of the mutant strain were prepared from glycerol and then used in the biotransformation of xylose into xylonate, an important platform chemical with versatile applications [30, 31].

## Results and discussion

### *G. oxydans* 621H can grow in mineral salts media with glycerol

**Fig. 2:**
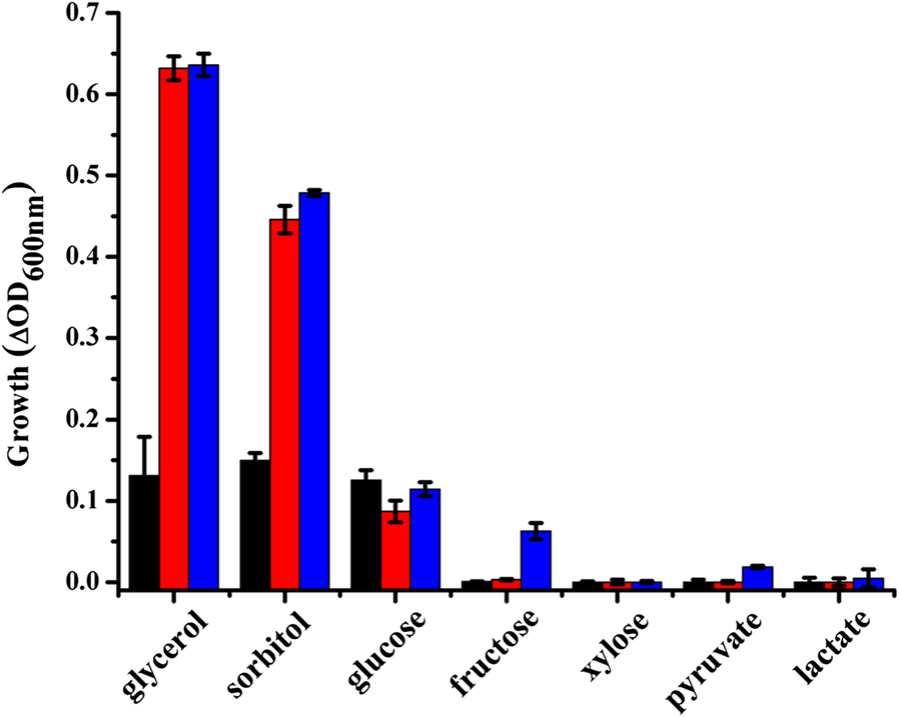
Growth of *G. oxydans* 621H in mineral salts media containing 10 g L^−1^ of different carbon sources. Growth (ΔOD_600 nm_) was calculated as the final OD reached at 12 h (black); 24 h (red); and 36 h (blue) minus the initial OD at 600 nm. The error bars represent the standard deviations of three independent experiments

Up to 73 g L^−1^ sorbitol is usually required for the growth of *G. oxydans* in complex media [32]. In order to reduce the culturing costs, the growth of *G. oxydans* 621H in mineral salts media containing 10 g L^−1^ various carbon sources including glycerol, sorbitol, glucose, fructose, xylose, pyruvate, and lactate was firstly studied. As shown in Fig. 2, *G. oxydans* 621H can grow in mineral salts media with glycerol, sorbitol, or glucose as the sole carbon source. After culture for 24 h, ∆OD was 0.63 when *G. oxydans* 621H was grown in glycerol, which was higher than that of the strain cultured in sorbitol and glucose. Growth of *G. oxydans* in medium containing glucose proceeds in two metabolic phases [33]. The fluctuation of ∆OD when *G. oxydans* 621H was grown in glucose might be due to the switch of different metabolic phases.

### There are three potential pathways related to glycerol metabolism

**Fig. 3:**
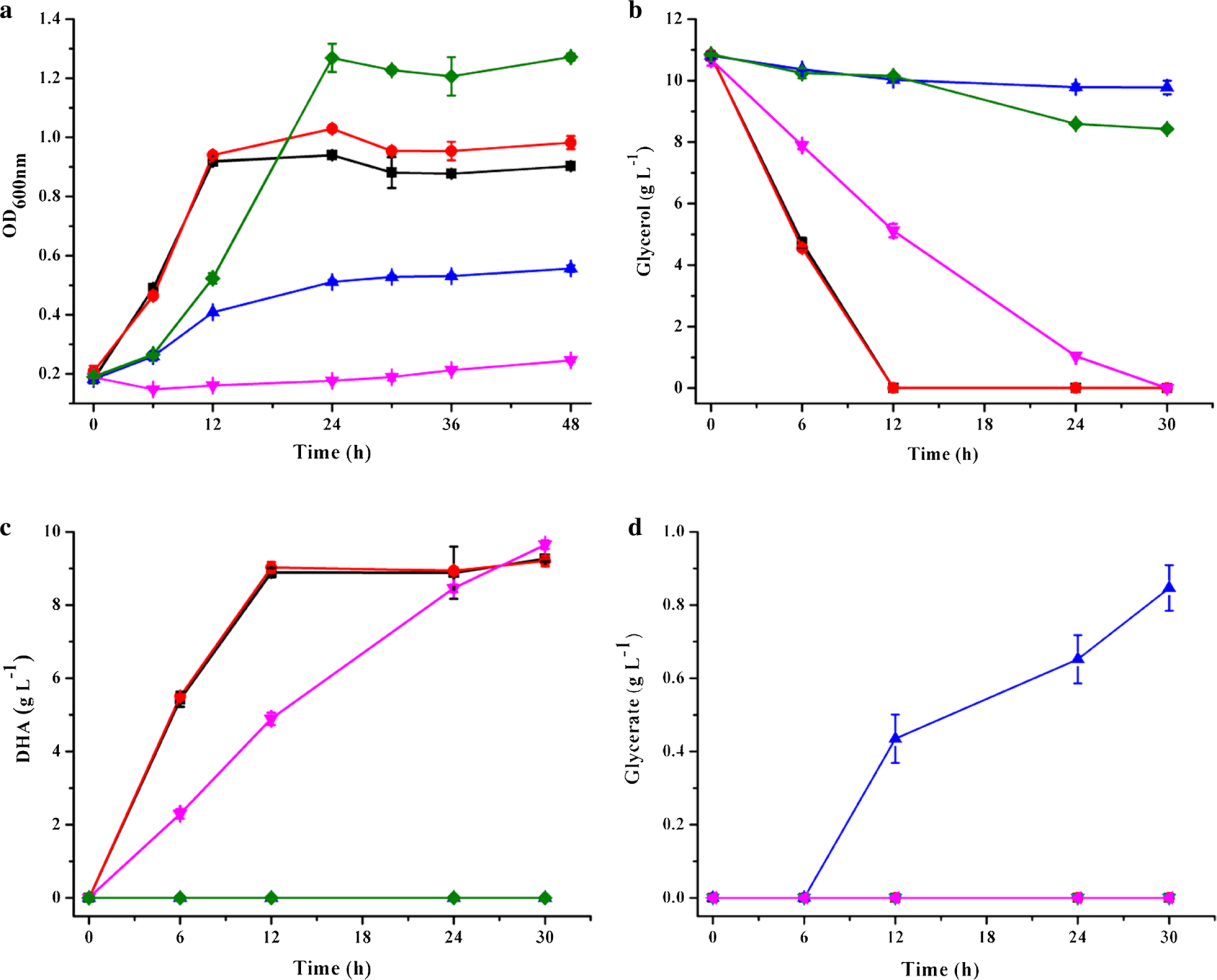
Growth of *G. oxydans* 621H and its derivatives in mineral salts medium with 10 g L^−1^ of glycerol as the sole carbon source. Cell density **a**, glycerol **b**, DHA **c**, and glycerate **d** were assayed. (Black square) *G. oxydans* 621H, (red circle) *G. oxydans* ∆GOX1068, (blue triangle) *G. oxydans* ∆GOX0854, (pink inverted triangle) *G. oxydans* ∆GOX2088 and (green diamond) *G. oxydans* ∆GOX1068∆GOX0854. The error bars represent the standard deviations of three independent experiments

The robust growth of *G. oxydans* 621H on glycerol was accompanied by the rapid consumption of glycerol and the accumulation of a large amount of DHA and a trace amount of glycerate (Fig. 3). After 24 h of culture, 10.8 ± 0.07 g L^−1^ of glycerol were consumed and the OD_600 nm_ of *G. oxydans* reached around 0.9. DHA gradually accumulated to a final concentration of 9.26 ± 0.07 g L^−1^. The yield of DHA was 0.86 g g^−1^ glycerol, indicating that glycerol was majorly oxidized to DHA during the growth of *G. oxydans* 621H.

As shown in Table 1, three putative enzymatic systems related to glycerol metabolism have been annotated in the genome of *G. oxydans* 621H [34]. Glycerol can be catalyzed to glyceraldehyde by membrane-bound alcohol dehydrogenase that consists of two subunits (GOX1067 and GOX1068), and then glyceraldehyde will be oxidized to glycerate by aldehyde dehydrogenase. Glycerol can also be converted into DHA by the major polyol dehydrogenase that consists of two subunits, GOX0854 and GOX0855. There is also a glycerol utilization operon that consists of *glpD* encoding a glycerol-3-phosphate dehydrogenase (GOX2088), *glpF* encoding a glycerol transporter (GOX2089), *glpK* encoding a glycerol kinase (GOX2090), and *glpR* encoding a transcriptional regulatory protein (GOX2087). Glycerol can be transported into cells through GlpF, phosphorylated by GlpK to produce glycerol-3-phosphate (G3P) and G3P will then be dehydrogenated by GlpD to dihydroxyacetone phosphate (DHAP). DHAP will finally be channeled into central metabolism [35–37].

**Table 1:** Genes related to glycerol utilization in *G. oxydans* 621H

Gene	Locus tag	Protein	Function
*adhAB*	GOX1067-1068	Alcohol dehydrogenase cytochrome c subunit	Dehydrogenation of primary alcohols
*sldAB*	GOX0854-0855	Glycerol dehydrogenase	Oxidation of glycerol to DHA
*glpR*	GOX2087	Glycerol-3-phosphate regulon repressor	Transcription regulation
*glpD*	GOX2088	Glycerol-3-phosphate dehydrogenase	Oxidation of G3P to DHAP
*glpF*	GOX2089	Glycerol uptake facilitator protein	Transporter activity
*glpK*	GOX2090	Glycerol kinase	Phosphorylation of glycerol to G3P

### GlpD is indispensable for glycerol metabolism of *G. oxydans* 621H

To identify the roles of these three enzymatic systems in glycerol metabolism and biomass production of *G. oxydans* 621H, four mutant strains including *G. oxydans* ∆GOX1068, *G. oxydans* ∆GOX0854, *G. oxydans* ∆GOX2088, and *G. oxydans* ∆GOX1068∆GOX0854 were constructed (Additional file 2: Fig. S1). *G. oxydans* ∆GOX0854 lost the ability to produce DHA, confirming that the major polyol dehydrogenase is responsible for DHA production. *G. oxydans* ∆GOX1068 produced 0.85 ± 0.06 g L^−1^ of glycerate but *G. oxydans* ∆GOX1068∆GOX0854 could not produce any glycerate, indicating that the membrane-bound alcohol dehydrogenase is responsible for glycerate production. As shown in Fig. 3a, only *G. oxydans* ∆GOX2088 could not grow in mineral salts medium with glycerol. Thus, the glycerol utilization operon is the sole system that is indispensable for biomass production from glycerol. On the other hand, the cells of *G. oxydans* ∆GOX2088 inoculated in the medium had polyol dehydrogenase activity and thus could consume glycerol and produce DHA with a low rate.

Membrane-bound dehydrogenases are necessary for the utilization of certain substrates as the sole carbon source only if the substrate cannot be assimilated directly by *G. oxydans*. For example, *G. oxydans* can not directly utilize mannitol. *G. oxydans* first oxidizes mannitol into fructose by the membrane-bound polyol dehydrogenase, then fructose can serve as a direct carbon source for the organism [38]. As shown in Fig. 3a, mutant of the membrane-bound dehydrogenases (the major polyol dehydrogenase and the alcohol dehydrogenase) only decreased the growth rate of *G. oxydans* 621H, but the biomass yield of *G. oxydans* ∆GOX1068∆GOX0854 was higher than other strains when glycerol was used as the sole carbon source.

The presence of membrane-bound dehydrogenases can increase the growth rate of *G. oxydans* 621H through efficient energy generation by feeding electrons from the glycerol oxidation directly into electron transport chain. However, the glycerol oxidation process would also decrease the amount of glycerol that can enter the central metabolic pathway through glycerol utilization operon. *G. oxydans* 621H, *G. oxydans* ∆GOX1068, *G. oxydans* ∆GOX0854, and *G. oxydans* ∆GOX1068∆GOX0854 were cultured in mineral salts medium with glycerol for 48 h, then the yields of biomass and products were assayed (Table 2). The biomass yield of *G. oxydans* ∆GOX1068∆GOX0854 from glycerol was 0.199 ± 0.018 g dry cell weight (DCW) g^−1^, which was higher than that of other strains. No production of DHA and glycerate could be detected during the culture of *G. oxydans* ∆GOX1068∆GOX0854. Thus, preparing whole cells of *G. oxydans* ∆GOX1068∆GOX0854 as the biocatalyst from glycerol may be more economic feasible than other strains.

**Table 2:** Biomass and product yields of *G. oxydans* and its derivatives in mineral salts medium with 10 g L^−1^ of glycerol as the sole carbon source

Strain	Yield of cell (g DCW g^−1^)	Yield of DHA (mol mol^−1^)	Yield of glycerate (mol mol^−1^)
WT	0.029 ± 0.001	0.987 ± 0.031	0.006 ± 0.0
∆GOX2088	0.003 ± 0.001	1.072 ± 0.021	0.012 ± 0.001
∆GOX1068	0.032 ± 0.001	0.994 ± 0.046	0.002 ± 0.0
∆GOX0854	0.069 ± 0.005	0.0 ± 0.0	0.744 ± 0.039
∆GOX1068∆GOX0854	0.199 ± 0.018	0.0 ± 0.0	0.0 ± 0.0

Data are the mean ± standard deviations (SDs) from three parallel experiments. The yields of DHA, glycerate and biomass of *G. oxydans* and its derivatives were calculated based on concentrations of the products and consumed glycerol

### *G. oxydans* ∆GOX1068∆GOX0854 has membrane-bound glucose dehydrogenase activity

Eight known and at least two unknown membrane-bound dehydrogenases genes have been annotated in the genome of *G. oxydans* 621H [38, 39]. These membrane-bound dehydrogenases can oxidize a wide range of compounds and can be used in various biocatalysis processes. Although *G. oxydans* ∆GOX1068∆GOX0854 lost two membrane-bound dehydrogenases catalyzing DHA and glycerate production, whole cells of the strain might be used in other chemicals production. For instance, the membrane-bound glucose dehydrogenase (GOX0265) has the ability to oxidize glucose, xylose, galactose, mannose, allose, and arabinose [38–40]. To identify the applicability of the whole cells of *G. oxydans* ∆GOX1068∆GOX0854 prepared from glycerol, the membrane-bound glucose dehydrogenase catalyzed xylose oxidation was selected as an example for further study.

Dehydrogenase activities toward glucose and xylose in crude cell extracts of *G. oxydans* 621H and *G. oxydans* ∆GOX1068∆GOX0854 were assayed. As shown in Table 3, the glucose dehydrogenase activities of *G. oxydans* 621H cultured in the traditional complex medium with 18.4 g L^−1^ of yeast extract and 73 g L^−1^ of sorbitol (Y-S medium) toward glucose and xylose were similar to that of *G. oxydans* ∆GOX1068∆GOX0854 cultured in mineral salts medium with 10 g L^−1^ of glycerol (M-G medium).

**Table 3:** Enzymatic activities of *G. oxydans* 621H and *G. oxydans* ∆GOX1068∆GOX0854 cultured in different media

Strain	Dehydrogenase activity towards glucose (mU mg^−1^)		Dehydrogenase activity towards xylose (mU mg^−1^)	
	Y-S	M-G	Y-S	M-G
WT	667.38 ± 23.83	260.95 ± 8.92	136.76 ± 20	54.49 ± 4.63
∆GOX1068∆GOX0854	894.73 ± 53.81	641.88 ± 2.95	127.06 ± 2.48	79.81 ± 9.24

Data are the mean ± standard deviations (SDs) from three parallel experiments

Y-S medium, complex medium with 18.4 g L^−1^ of yeast extract and 73 g L^−1^ of sorbitol

M-G medium, mineral salts medium with 10 g L^−1^ of glycerol

### Whole cells of *G. oxydans* ∆GOX1068∆GOX0854 can be prepared with low glycerol consumption

**Fig. 4:**
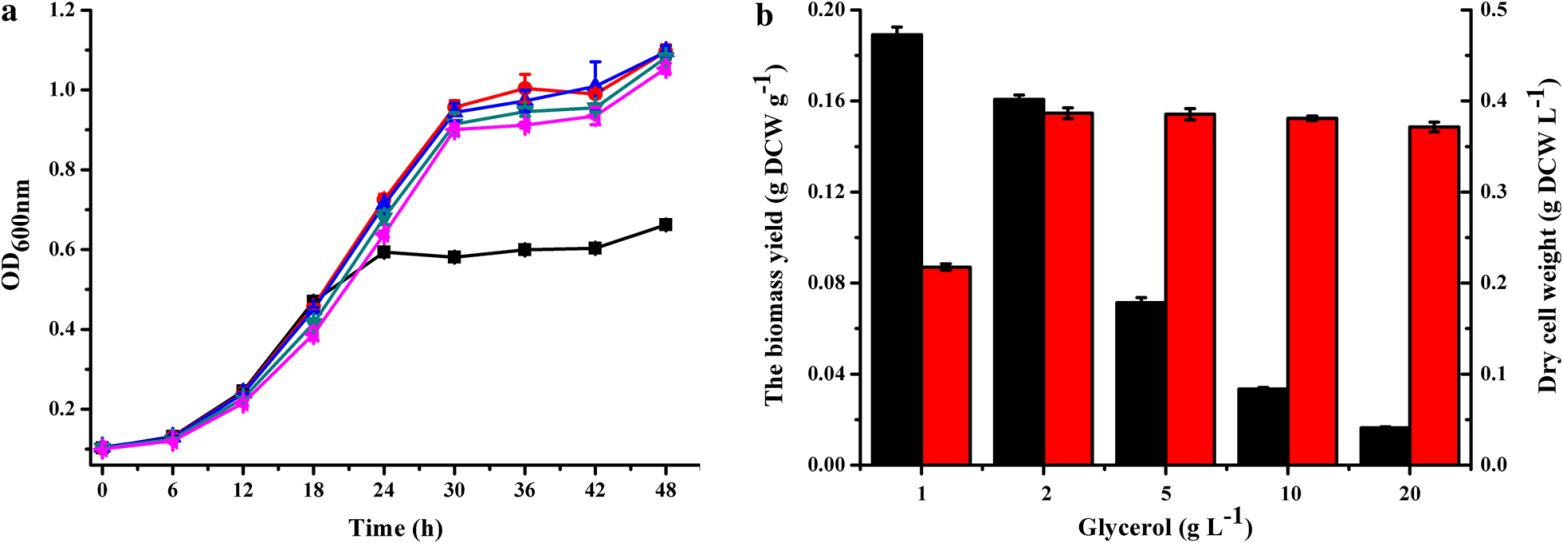
Growth of *G. oxydans* ∆GOX1068∆GOX0854 in mineral salts medium containing different concentrations of glycerol. **a** Cell density, (black square) 1 g L^−1^, (red circle) 2 g L^−1^, (blue triangle) 5 g L^−1^, (green inverted triangle) 10 g L^−1^, (pink left-pointing triangle) 20 g L^−1^. Consumption of glycerol could be found in Additional file 3: Figure S2. **b** The dry cell weight (red) and biomass yield (black) of *G. oxydans* ∆GOX1068∆GOX0854 in mineral salts medium containing different concentrations of glycerol. The yield of *G. oxydans* ∆GOX1068∆GOX0854 was calculated based on concentrations of the cells and added glycerol. Calculation of dry cell weight: g DCW L^−1^ = 0.3896 × ∆OD_600 nm _− 0.0004. The error bars represent the standard deviations of three independent experiments

When *G. oxydans* ∆GOX1068∆GOX0854 was cultured in mineral salts medium with 10 g L^−1^ of glycerol, the glycerol consumed is less than 2 g L^−1^ (Fig. 3b). Thus, *G. oxydans* 621H and *G. oxydans* ∆GOX1068∆GOX0854 were cultured in mineral salts medium with different concentrations of glycerol and the growth of the strains was assayed (Additional file 3: Fig. S2). *G. oxydans* ∆GOX1068∆GOX0854 could grow to similar OD_600 nm_ and exhibited similar dehydrogenase activity toward xylose in mineral salts medium with 2, 5, 10, and 20 g L^−1^ of glycerol (Fig. 4a and Additional file 4: Fig. S3). The culture system with 2 g L^−1^ of glycerol as the substrate exhibited relatively high biomass yield (Fig. 4b). Under identical conditions, the biomass yield of *G. oxydans* ∆GOX1068∆GOX0854 (0.161 ± 0.002 g DCW g^−1^) was 2.4-fold higher than that of *G. oxydans* 621H (0.067 ± 0.002 g DCW g^−1^) (Additional file 5: Fig. S4). Whole cells of *G. oxydans* ∆GOX1068∆GOX0854 can be prepared with a low glycerol consumption (2 g L^−1^) and may decrease the biocatalyst preparation cost.

### Xylonate can be produced from xylose using whole cells of *G. oxydans* ∆GOX1068∆GOX0854

**Fig. 5:**
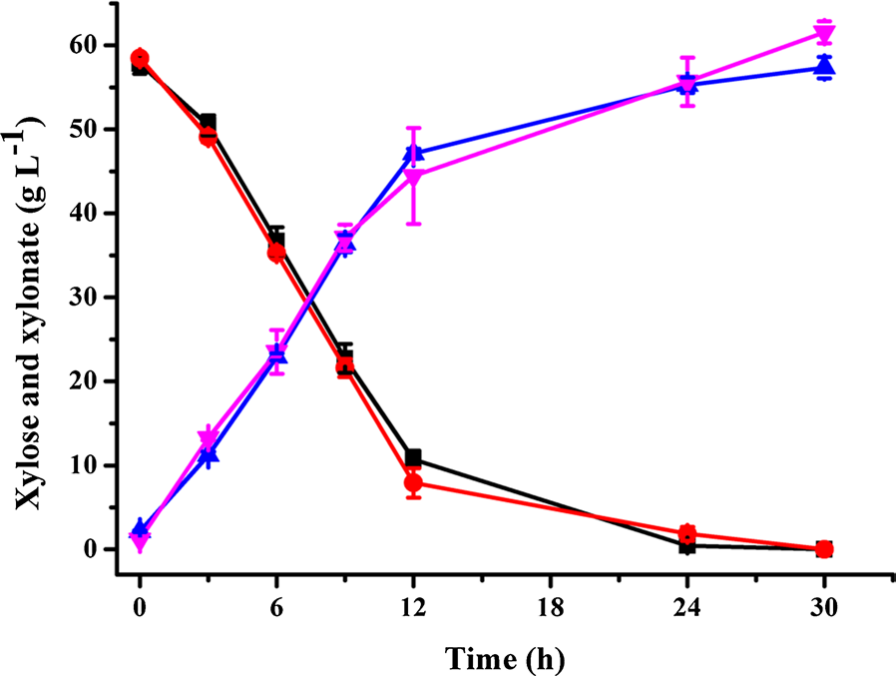
Conversion of xylose into xylonate using whole cells of *G. oxydans* 621H and *G. oxydans* ∆GOX1068∆GOX0854 prepared from the mineral salts medium with 2 g L^−1^ glycerol as the sole carbon source. (Black square) xylose consumption of *G. oxydans* 621H, (red circle) xylose consumption of *G. oxydans* ∆GOX1068∆GOX0854, (blue triangle) xylonate formation of *G. oxydans* 621H, (pink inverted triangle) xylonate formation of *G. oxydans* ∆GOX1068∆GOX0854. The error bars represent the standard deviations of three independent experiments

Production of xylonate from xylose was used to assay the biocatalytic performance of whole cells of *G. oxydans* 621H and *G. oxydans* ∆GOX1068∆GOX0854 prepared from 2 g L^−1^ glycerol. Biocatalysis was conducted at 30 °C in 20 mM potassium phosphate buffer (pH was adjusted to 7.0) with 60 g L^−1^ of xylose as the substrate and 7.8 g DCW L^−1^ of whole cells of *G. oxydans* 621H and *G. oxydans* ∆GOX1068∆GOX0854 as the biocatalysts. As shown in Fig. 5, after 30 h of biotransformation, up to 61.6 g L^−1^ xylonate was produced at a yield of 1.05 g g^−1^ by *G. oxydans* ∆GOX1068∆GOX0854. The specific yield of xylonate per unit cells was 7.90 g xylonate/g DCW. Xylonate at a final concentration of 57.4 g L^−1^ was produced with a yield of 0.99 g g^−1^ by *G. oxydans* 621H. The specific yield of xylonate per unit cells was 7.36 g xylonate/g DCW.

**Fig. 6:**
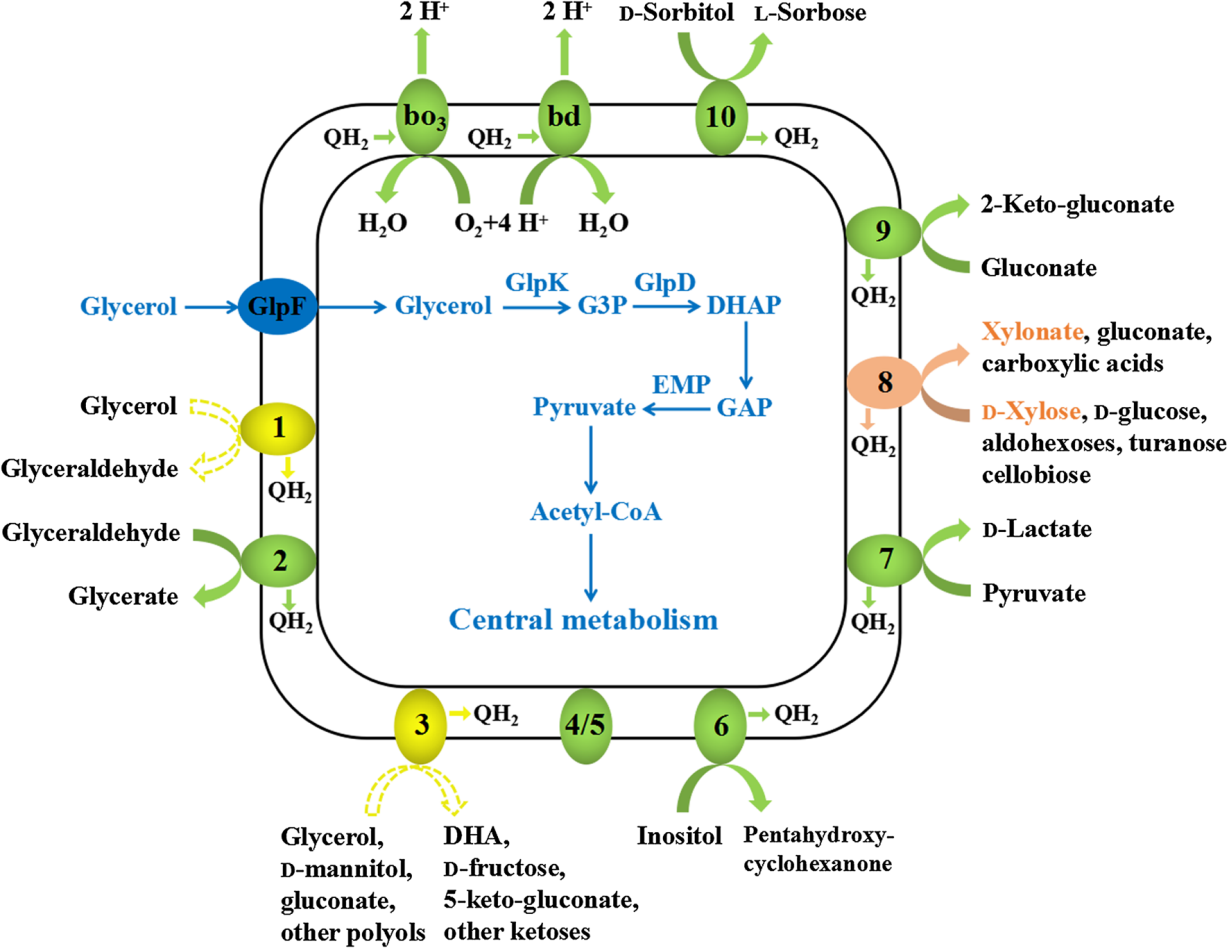
Scheme of xylonate production using metabolic engineered *G. oxydans* 621H. The pathway marked with blue color indicated the glycerol utilization pathway used for biomass production. The pathways marked with yellow color were deleted to increase the glycerol channeled into central metabolism. The pathway marked with pink color indicated the xylonate production from xylose using the membrane-bound glucose dehydrogenase. The pathways marked with green color indicated remaining membrane-bound dehydrogenases could be used in other oxidative biotransformation processes. *bo*_*3*_, cytochrome *bo*_*3*_ ubiquinol oxidase, *bd*, cytochrome *bd* ubiquinol oxidase. Membrane-bound dehydrogenases transfer electrons to ubiquinone to form the reduced cofactor ubiquinol (QH_2_), which is oxidized by *bo*_*3*_ and *bd* to generate an electron gradient for energy production. 1, alcohol dehydrogenase (GOX1067 and GOX1068); 2, aldehyde dehydrogenase (GOX0585, GOX0586 and GOX0587); 3, polyol dehydrogenase (GOX0854 and GOX0855); 4/5, uncharacterized PQQ-depending dehydrogenase; 6, inositol dehydrogenase (GOX1857); 7, d-lactate dehydrogenase (GOX1253); 8, glucose dehydrogenase (GOX0265); 9, gluconate dehydrogenase (GOX1230, GOX1231 and GOX1232); 10, sorbitol dehydrogenase (GOX2094, GOX2095, GOX2096 and GOX2097). *DHA* dihydroxyacetone, *G3P* glycerol-3-phosphate, *DHAP* dihydroxyacetone phosphate, *GAP* glyceraldehyde-3-phosphate, *GlpF* glycerol uptake facilitator protein, *GlpK* glycerol kinase, *GlpD* glycerol-3-phosphate dehydrogenase, *EMP* Embden–Meyerhof–Parnas pathway

Typically, whole cells of *G. oxydans* could be used as the biocatalysts in the industrial applications. Increased biomass yields from carbon sources used would lower the production costs of the biocatalysts. Therefore, various studies have aimed at increases of the biomass yields of *G. oxydans* 621H in complex media by metabolic engineering [9, 10, 41]. This study was the first study that focused on the construction of *G. oxydans* to lower the production cost in mineral salts medium. Glycerol, a chemical generated during the biofuel production, is a comparably cheap carbon source that can support the growth of *G. oxydans* 621H in mineral salts medium. Three enzymes’ systems related to glycerol metabolism including the major polyol dehydrogenase, the membrane-bound alcohol dehydrogenase, and the glycerol-3-phosphate dehydrogenase were predicted in genome of *G. oxydans* 621H [34]. Their possible biological functions have been tentatively studied through gene deletion (Fig. 6). Complementation of these genes in the related mutant strains might further demonstrate their functions in the glycerol metabolism of *G. oxydans* 621H.

The major polyol dehydrogenase and the membrane-bound alcohol dehydrogenase play no obvious roles in biocatalyst preparation but can decrease the glycerol channeled into biomass production. Thus, these two enzymes were deleted and the result strain *G. oxydans* ∆GOX1068∆GOX0854 only has the glycerol utilization operon and can efficiently transform glycerol into biomass with high yield and low glycerol consumption (Fig. 6). Eight known and two unknown membrane-bound dehydrogenases were predicted in *G. oxydans* 621H [38, 39]. Except to the major polyol dehydrogenase and the membrane-bound alcohol dehydrogenase involved in DHA and glycerate production, the activity of the membrane-bound dehydrogenases involved in other desired industrial applications might not be interfered. For example, production of xylonate from xylose with the membrane-bound glucose dehydrogenase was achieved using whole cells of the mutant strain prepared from glycerol. This biocatalyst might also be used in other oxidative biotransformation processes depending on the membrane-bound dehydrogenases of the strain.

## Conclusions

In this study, we develop a biocatalyst preparation process for *G. oxydans* 621H using biofuel’s byproduct glycerol as the carbon source in mineral salts medium. Two glycerol oxidation enzymes were deleted in *G. oxydans* 621H and the double knock-out strain could utilize glycerol with a higher biomass yield than that of the wild-type strain. Using the whole cells of the mutant strain as the biocatalyst, 61.6 g L^−1^ xylonate was produced from xylose in 30 h with high yield and high productivity. This research provides a low-cost and a renewable strategy for low cost production of whole-cell biocatalyst of *G. oxydans*, which may be used in various biocatalytic processes.

## Methods

### Bacterial strains and culture conditions

The strains, plasmids and primers used in this study are listed in Table 4. *G. oxydans* 621H and its derivatives were cultured in a complex medium containing 73 g sorbitol, 18.4 g yeast, 1.5 g (NH_4_)_2_SO_4_, 1.5 g KH_2_PO_4_ and 0.47 g MgSO_4_·7H_2_O in 1 L of distilled water. A mineral salt medium containing 0.2 g l-glutamine, 2 g (NH_4_)_2_SO_4_, 0.2 g MgSO_4_·7H_2_O, 2.2 g KH_2_PO_4_, 0.2 g Na_2_HPO_4_, 5 mg nitrilotriacetic acid, 30 mg EDTA, 11 mg FeSO_4_·7H_2_O, 9 mg ZnSO_4_·7H_2_O, 0.6 mg CoCl_2_·6H_2_O, 2 mg MnCl_2_·4H_2_O, 0.6 mg CuSO_4_·5H_2_O, 9 mg CaCl_2_·2H_2_O, 0.08 mg NaMoO_4_·2H_2_O, 1 mg H_3_BO_3_, 0.2 mg KI, 0.5 mg calcium pantothenate, 0.4 mg nicotinic acid, 0.4 mg *p*-aminobenzoic acid, and different carbon sources in 1 L of distilled water were also used [42]. *G. oxydans* strains were grown at 30 °C and 200 rpm in 250 mL baffled Erlenmeyer flasks. *E. coli* strains were cultivated at 37 °C in Luria–Bertani medium (yeast 5 g L^−1^, tryptone 10 g L^−1^, NaCl 10 g L^−1^) at 37 °C and 180 rpm on a rotary shaker. Antibiotics were used when necessary at the following concentrations: kanamycin at 50 μg mL^−1^, ampicillin at 100 μg mL^−1^ and 50 μg mL^−1^ cefoxitin.

**Table 4:** Strains, plasmids and primers used in this study

Name	Relevant characteristic^a^	Reference
Strain		
*G. oxydans* 621H	*G. oxydans* DSM2343, wild type, Cef^r^	DSMZ^b^
∆GOX1068	*gox1068* deletion strain of *G. oxydans* 621H	This study
∆GOX0854	*gox0854* deletion strain of *G. oxydans* 621H	This study
∆GOX1068∆GOX0854	*gox1068* and *gox0854* deletion strain of *G. oxydans* 621H, Cef^r^	This study
∆GOX2088	*gox2088* deletion strain of *G. oxydans* 621H	This study
*E. coli* DH5α	F^−^ *supE44* ∆*lacU169* (*φ80 lacZ*∆*M15*) *hsdR17 recA1 endA1 gyrA96 thi*^−*1*^ *relA1 λ*^−^	Novagen
*E. coli* HB101	*F*^*−*^ *mcrB mrr hsdS20(rB*^−^*mB*^*−*^*) recA13 supE44 ara14 galK2 lacY1 proA2 rpsL20(Sm*^*r*^*) xy15 λ*^*-*^*leu mtl1;* used for triparental mating as the helper strain	Invitrogen
Plasmid		
pK18*mobsacB*	Suicide plasmid, *ori*ColE1 Mob^+^, *lacZα*, *sacB*, Km^r^	Schäfer et al.
pK18*mobsacB*-∆GOX1068	pK18*mobsacB* with partial *gox1068* of *G. oxydans* 621H for deletion	This study
pK18*mobsacB*-∆GOX0854	pK18*mobsacB* with partial *gox0854* of *G. oxydans* 621H for deletion	This study
pK18*mobsacB*-∆GOX1068∆GOX0854	pK18*mobsacB* with partial *gox1068* and *gox0854* of *G. oxydans* 621H for deletion	This study
pK18*mobsacB*-∆GOX2088	pK18*mobsacB* with partial *gox2088* of *G. oxydans* 621H for deletion	This study
Primers		
QGOX1068.f	CCAGAATTCGATGACTTCTGGTCTACTGAC (*EcoR*I)	This study
QGOX1068.r	TAAGGATCCTCAGGGGTGATCCGCGGTCG (*BamH*I)	This study
GOX1068up.r.	GAGCAGAACGTCTGAGTTGGTCGTGGCGTA	This study
GOX1068down.f	TACGCCACGACCAACTCAGACGTTCTGCTC	This study
QGOX0854.f	CCAGAATTCGATGCGCAGATCCCATCTTCT (*EcoR*I)	This study
QGOX0854.r	TATAAGCTTTCAGCCCTTGTGATCAGGCA (*Hind*III)	This study
GOX0854up.r.	GCCCCACGGACCATTCTGGCACTCTGCTGC	This study
GOX0854down.f	GCAGCAGAGTGCCAGAATGGTCCGTGGGGC	This study
GOXQ2088.f	CGCGGATCCATGTCATCGATTCACGAGGTTTCA (*BamH*I)	This study
QGOX2088.r	CCCAAGCTTTCAGGCGACGTTCTGACGTGCGA (*Hind*III)	This study
GOX2088up.r.	CGAAAACCGACAGGAAGCCGGCTGTCCTGC	This study
GOX2088down.f	GCAGGACAGCCGGCTTCCTGTCGGTTTTCG	This study

^a^Cef^r^ and Km^r^ were represented Cefoxitin resistance and Kanamycin resistance, respectively

^b^DSMZ, Deutsche Sammlung von Mikroorganismen und Zellkulturen, Braunschweig, Germany

### General molecular biological techniques

Molecular biological techniques were carried out using standard protocols [43]. Genomic DNA of *G. oxydans* 621H was extracted through the Wizard Genomic DNA Purification Kit (Promega, Madison, WI, USA). Polymerase chain reaction (PCR) primers were obtained from Sangon (Shanghai, China). Restriction enzymes were obtained from Thermo Fisher Scientific (USA). T_4_ DNA ligase and fastPfu DNA polymerase were purchased from MBI (USA) and Transgen Biotech (China), respectively. *G. oxydans* 621H was transformed via a triparental mating method as described previously [39, 44]. All the constructed strains were validated by DNA sequencing done by Sangon (Shanghai, China).

### Deletion of genes in *G. oxydans* 621H

Genes of *G. oxydans* 621H were deleted using the pK18*mobsacB* system as described previously [45]. Briefly, the homologous arms upstream and downstream of the target GOX1068 gene were amplified by PCR with the genomic DNA of *G. oxydans* 621H as the template, and the primers GOX1068up.f/GOX1068up.r and GOX1068down.f/GOX1068down.r. Then they were fused together by the recombinant PCR using primers GOX1068up.f/GOX1068down.r, which contained *Eco*RI and *Bam*HI restriction enzyme sites, respectively (Table 4). The generated fusion construct and pK18*mobsacB* [46], a mobilizable plasmid that does not replicate in *G. oxydans*, were digested with *Eco*RI and *Bam*HI, and then linked by using T4 DNA ligase to form pK18*mobsacB*-∆GOX1068. The plasmid was transferred into *G. oxydans* via a triparental mating method [44]. *E. coli* DH5α harboring pK18*mobsacB*-∆GOX1068 was used as the donor strain, *E. coli* HB101 harboring the plasmid pRK2013 was used as the helper strain, and *G. oxydans* 621H was used as the recipient strain. The first crossover cells containing the integration of the plasmid pK18*mobsacB*-∆GOX1068 into the chromosome of *G. oxydans* 621H were selected on the complex medium agar plate supplemented with 50 μg mL^−1^ kanamycin, 50 μg mL^−1^ cefoxitin, and 0.1% (vol/vol) acetic acid [45]. The second crossover cells were singled out by culture on the complex medium plates containing 10% (w/v) sucrose. Other mutants of *G. oxydans* 621H were generated by using the same procedure and primers listed in Table 4. All mutants were verified by PCR and sequencing.

### Enzymatic activity assays

The oxidation of glucose and xylose in *G. oxydans* 621H was majorly catalyzed by membrane-bound glucose dehydrogenase, which can be assayed using DCPIP as the electron acceptor [38]. Cells of *G. oxydans* 621H and its derivatives were collected, washed, and then resuspended in phosphate-buffered saline (pH 7.4 PBS) supplemented with 10% v/v glycerol and 1.0 mM phenylmethylsulfonyl fluoride (PMSF), and lysed by sonication on ice. Cell debris was removed through centrifugation (13,000 rpm, 20 min). The activities of membrane bound glucose dehydrogenase were assayed by monitoring the change in absorbance at 600 nm corresponding to the reduction of DCPIP with a UV/visible spectrophotometer (Ultrospec 2100 pro, Amersham Biosciences, USA). The reaction was carried out in 0.8 mL of pH 7.4 PBS, containing 20 mM PMS, 50 mM DCPIP, 25 mM glucose or xylose and 40 μL crude cell extracts.

### Production of xylonate from xylose through whole-cell biotransformation

*G. oxydans* ΔGOX1068∆GOX0854 were cultivated in mineral salts medium with 2 g L^−1^ glycerol at 30 °C and 200 rpm for 36 h. The cells were harvested, washed twice with PBS (pH 7.4), and then resuspended in the same buffer. The biotransformation reactions were carried out at 30 °C and 150 rpm in 20 mM potassium phosphate buffer (pH was adjusted to 7.0) containing 7.8 g DCW L^−1^ of whole cells of *G*. *oxydans* 621H or *G. oxydans* ∆GOX1068∆GOX0854 and 60 g L^−1^ of xylose. Samples were taken every 3 h and centrifuged at 13,000 rpm for 5 min before being analyzed.

### Analytical methods

The biomass was evaluated by determination of optical density (OD) at 600 nm with a UV/visible spectrophotometer. Optical densities of whole cells were converted to DCW as Y = 0.3896 × OD_600 nm _− 0.0004. The protein concentration was determined by the Lowry procedure using bovine serum albumin as the standard [47].

The samples obtained from the biotransformation experiment were filtered through a 0.22 μm filter prior to high-performance liquid chromatography (HPLC) analysis. Glycerol, glycerate, and DHA were quantified using an Aminex HPX-87H column (300 × 7.8 mm; Bio-Rad, USA) equipped with a RID-10A refractive index detector. Xylonate was quantified by an Aminex HPX-87H column (300 × 7.8 mm; Bio-Rad, USA) equipped with a SPD-M20A photodiode array detector at 210 nm. The analysis was carried out at 55 °C using 10 mM H_2_SO_4_ as the eluent at a flow rate of 0.4 mL min^−1^. Xylose was analyzed using an Aminex HPX-87P column (300 × 7.8 mm; Bio-Rad, USA) equipped with a RID-10A refractive index detector at 65 °C and the mobile phase was deionized water at a flow rate of 0.6 mL min^−1^. A series of injections of standard substances in the range of 0–10 g L^−1^ was used to obtain calibration curves.

## Additional files

**Additional file 1: Table S1.** Price comparison of different carbon sources.

**Additional file 2: Fig. S1.** PCR confirmation of the disruption of genes. a. *adhB* (GOX1068); b. *sldA* (GOX0854); c.  *adhB* and *sldA* (GOX1068GOX0854); d.  *glpd* (GOX2088). M: 5K Marker; WT: the genomic DNA of wild-type *G. oxydans* 621H; +: pK18*mobsacB-*ΔGOX0854 (b), pK18*mobsacB*-ΔGOX2088 (d); -:H_2_O.

**Additional file 3: Fig. S2.** Consumption of substrate of *G. oxydans* ΔGOX1068ΔGOX0854 in mineral salts medium containing different concentrations of glycerol. (Black square) 1 g L^−1^, (red circle) 2 g L^−1^, (blue triangle) 5 g L^−1^, (green inverted triangle) 10 g L^−1^, (pink left-pointing triangle) 20 g L^−1^.

**Additional file 4: Fig. S3.** Dehydrogenase activity toward xylose of *G. oxydans* ΔGOX1068ΔGOX0854 cultured in mineral salts medium containing different concentrations of glycerol.

**Additional file 5: Fig. S4.** The dry cell weight and biomass yield of *G. oxydans* 621H in mineral salts medium containing different concentrations of glycerol. Dry cell weight (red), biomass yield (black). The biomass yield was calculated based on concentrations of the cells and added glycerol. Calculation of dry cell weight: g DCW L^−1^ = 0.3896 × ΔOD_600 nm _− 0.0004.

## Abbreviations

DHA: dihydroxyacetone
DHAP: dihydroxyacetone phosphate
G3P: glycerol-3-phosphate
GAP: glyceraldehyde-3-phosphate
*glpR*: glycerol-3-phosphate regulon repressor
*glpD*: glycerol-3-phosphate dehydrogenase
*glpF*: glycerol uptake facilitator protein
*glpK*: glycerol kinase
EMP: Embden–Meyerhof–Parnas Pathway
DCW: dry cell weight
PCR: polymerase chain reaction
OD_600 nm_: optical density at 600 nm
PMSF: phenylmethanesulfonyl fluoride
DCPIP: 2,6-dichloroindophenol
PBS: phosphate-buffered saline
HPLC: high-performance liquid chromatography
