# Supplementary material for: Engineering of glycerol utilization in Gluconobacter oxydans 621H for biocatalyst preparation in a low-cost way
Source: Microb Cell Fact. 2018 Oct 8;17:158. doi: 10.1186/s12934-018-1001-0 (PMC6174558; doi:10.1186/s12934-018-1001-0)
Supplement: Supplementary file 1 — Additional file 1: Table S1. Price comparison of different carbon sources. [file 12934_2018_1001_MOESM1_ESM.pdf]

Additional File 1

Engineering of glycerol utilization in *Gluconobacter oxydans* 621H for biocatalyst preparation in a low-cost way

Jinxin Yan[1], Jing Xu[1,3], Menghao Cao[1], Zhong Li[1], Chengpeng Xu[1], Xinyu Wang[1], Chunyu Yang[1], Ping Xu[2], Chao Gao[1], Cuiqing Ma[1*]

[1]State Key Laboratory of Microbial Technology & Shenzhen Research Institute, Shandong University, 27 Shanda South Road, Jinan 250100, People's Republic of China

[2]State Key Laboratory of Microbial Metabolism, Joint International Research Laboratory of Metabolic & Developmental Sciences, and School of Life Sciences & Biotechnology, Shanghai Jiao Tong University, 800 Dongchuan Road, Shanghai 200240, People's Republic of China

[3]Dong Ying Oceanic and Fishery Bureau, 206 Yellow River Road, Dongying 257091, People's Republic of China

***Corresponding Author**

Cuiqing Ma, E-mail: macq@sdu.edu.cn. Tel.: +86-531-88369463. Fax: +86-531-88369463.

Additional file 1: Table S1 Price comparison of different carbon sources

**Carbon sources**	**Grade Standard**	**Purity (%)**	**Price ($/ton)[a]**
Glycerol	Medicine Grade	99.5	500-570
Sorbitol	Food Grade	70	600-900
Glucose	Food Grade	100	400-600
Fructose	Food Grade	99	1,200-2,000
Xylose	Food Grade	100	800-1200
Pyruvate	Medicine Grade	99	12,000-25,000
Lactate	Food Grade	15-40	2,200-2,600

[a] Prices of carbon sources from Alibaba International Station
