# Supplementary material for: Engineering of glycerol utilization in Gluconobacter oxydans 621H for biocatalyst preparation in a low-cost way
Source: Microb Cell Fact. 2018 Oct 8;17:158. doi: 10.1186/s12934-018-1001-0 (PMC6174558; doi:10.1186/s12934-018-1001-0)
Supplement: Supplementary file 2 — Additional file 2: Fig. S1. PCR confirmation of the disruption of genes. a. adhB (GOX1068); b. sldA (GOX0854); c. adhB and sldA (GOX1068GOX0854); d. glpd (GOX2088). M: 5K Marker; WT: the genomic DNA of wild-type G. oxydans 621H; +: pK18mobsacB-ΔGOX0854 (b), pK18mobsacB-ΔGOX2088 (d); -:H2O. [file 12934_2018_1001_MOESM2_ESM.pdf]

Additional File 2

Engineering of glycerol utilization in *Gluconobacter oxydans* 621H for biocatalyst preparation in a low-cost way

Jinxin Yan[1], Jing Xu[1,3], Menghao Cao[1], Zhong Li[1], Chengpeng Xu[1], Xinyu Wang[1], Chunyu Yang[1], Ping Xu[2], Chao Gao[1], Cuiqing Ma[1*]

[1]State Key Laboratory of Microbial Technology & Shenzhen Research Institute, Shandong University, 27 Shanda South Road, Jinan 250100, People's Republic of China

[2]State Key Laboratory of Microbial Metabolism, Joint International Research Laboratory of Metabolic & Developmental Sciences, and School of Life Sciences & Biotechnology, Shanghai Jiao Tong University, 800 Dongchuan Road, Shanghai 200240, People's Republic of China

[3]Dong Ying Oceanic and Fishery Bureau, 206 Yellow River Road, Dongying 257091, People's Republic of China

***Corresponding Author**

Cuiqing Ma, E-mail: macq@sdu.edu.cn. Tel.: +86-531-88369463. Fax: +86-531-88369463.

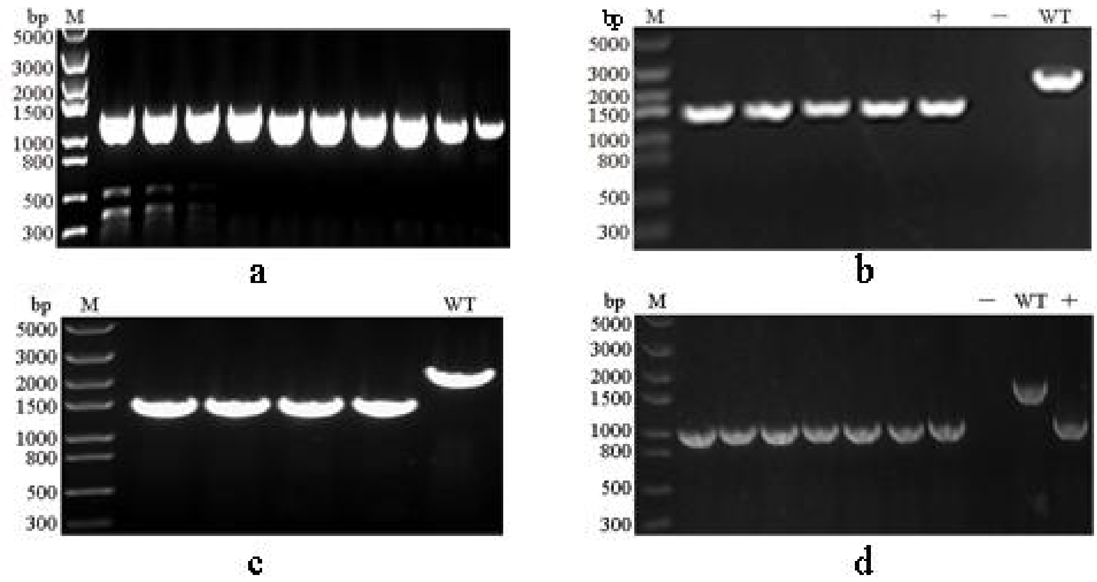

Additional file 2: Fig. S1 PCR confirmation of the disruption of genes. **a**. *adhB* (GOX1068); **b**. *sldA* (GOX0854); **c**. *adhB* and *sldA* (GOX1068GOX0854); **d**. *glpd* (GOX2088). M: 5K Marker; WT: the genomic DNA of wild-type *G. oxydans* 621H; ＋: pK18*mobsacB*-ΔGOX0854 (b), pK18*mobsacB*-ΔGOX2088 (d); －:H_2O.
