# Supplementary material for: Engineering of glycerol utilization in Gluconobacter oxydans 621H for biocatalyst preparation in a low-cost way
Source: Microb Cell Fact. 2018 Oct 8;17:158. doi: 10.1186/s12934-018-1001-0 (PMC6174558; doi:10.1186/s12934-018-1001-0)
Supplement: Supplementary file 3 — Additional file 3: Fig. S2. Consumption of substrate of G. oxydans ΔGOX1068ΔGOX0854 in mineral salts medium containing different concentrations of glycerol. (Black square) 1 g L−1, (red circle) 2 g L−1, (blue triangle) 5 g L−1, (green inverted triangle) 10 g L−1, (pink left-pointing triangle) 20 g L−1. [file 12934_2018_1001_MOESM3_ESM.pdf]

Additional File 3

Engineering of glycerol utilization in *Gluconobacter oxydans* 621H for biocatalyst preparation in a low-cost way

Jinxin Yan[1], Jing Xu[1,3], Menghao Cao[1], Zhong Li[1], Chengpeng Xu[1], Xinyu Wang[1], Chunyu Yang[1], Ping Xu[2], Chao Gao[1], Cuiqing Ma[1*]

[1]State Key Laboratory of Microbial Technology & Shenzhen Research Institute, Shandong University, 27 Shanda South Road, Jinan 250100, People's Republic of China

[2]State Key Laboratory of Microbial Metabolism, Joint International Research Laboratory of Metabolic & Developmental Sciences, and School of Life Sciences & Biotechnology, Shanghai Jiao Tong University, 800 Dongchuan Road, Shanghai 200240, People's Republic of China

[3]Dong Ying Oceanic and Fishery Bureau, 206 Yellow River Road, Dongying 257091, People's Republic of China

***Corresponding Author**

Cuiqing Ma, E-mail: macq@sdu.edu.cn. Tel.: +86-531-88369463. Fax: +86-531-88369463.

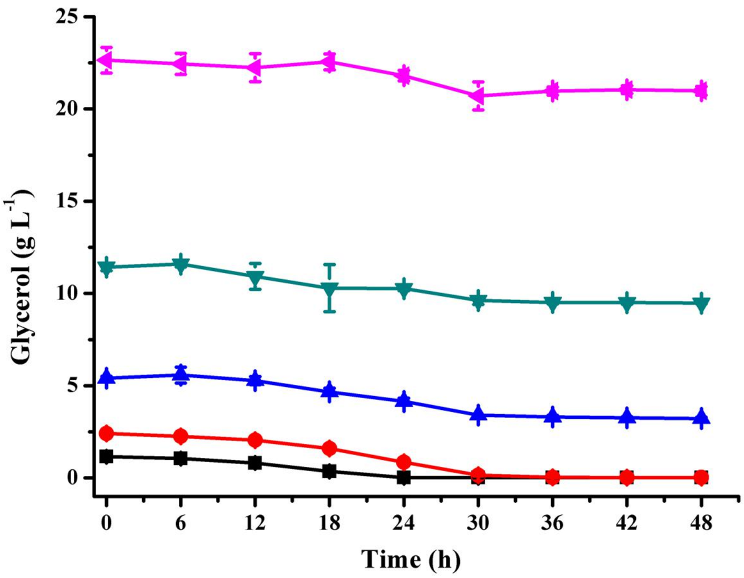

Additional file 3: Fig. S2 Consumption of substrate of *G. oxydans* ΔGOX1068ΔGOX0854 in mineral salts medium containing different concentrations of glycerol. (■) 1 g L^{-1}, (●) 2 g L^{-1}, (▲) 5 g L^{-1}, (▼) 10 g L^{-1}, (◀) 20 g L^{-1}.
