# Supplementary material for: Engineering of glycerol utilization in Gluconobacter oxydans 621H for biocatalyst preparation in a low-cost way
Source: Microb Cell Fact. 2018 Oct 8;17:158. doi: 10.1186/s12934-018-1001-0 (PMC6174558; doi:10.1186/s12934-018-1001-0)
Supplement: Supplementary file 5 — Additional file 5: Fig. S4. The dry cell weight and biomass yield of G. oxydans 621H in mineral salts medium containing different concentrations of glycerol. Dry cell weight (red), biomass yield (black). The biomass yield was calculated based on concentrations of the cells and added glycerol. Calculation of dry cell weight: g DCW L−1 = 0.3896 × ΔOD600 nm − 0.0004. [file 12934_2018_1001_MOESM5_ESM.pdf]

Additional File 5

Engineering of glycerol utilization in *Gluconobacter oxydans* 621H for biocatalyst preparation in a low-cost way

Jinxin Yan[1], Jing Xu[1,3], Menghao Cao[1], Zhong Li[1], Chengpeng Xu[1], Xinyu Wang[1], Chunyu Yang[1], Ping Xu[2], Chao Gao[1], Cuiqing Ma[1*]

[1]State Key Laboratory of Microbial Technology & Shenzhen Research Institute, Shandong University, 27 Shanda South Road, Jinan 250100, People's Republic of China

[2]State Key Laboratory of Microbial Metabolism, Joint International Research Laboratory of Metabolic & Developmental Sciences, and School of Life Sciences & Biotechnology, Shanghai Jiao Tong University, 800 Dongchuan Road, Shanghai 200240, People's Republic of China

[3]Dong Ying Oceanic and Fishery Bureau, 206 Yellow River Road, Dongying 257091, People's Republic of China

***Corresponding Author**

Cuiqing Ma, E-mail: macq@sdu.edu.cn. Tel.: +86-531-88369463. Fax: +86-531-88369463.

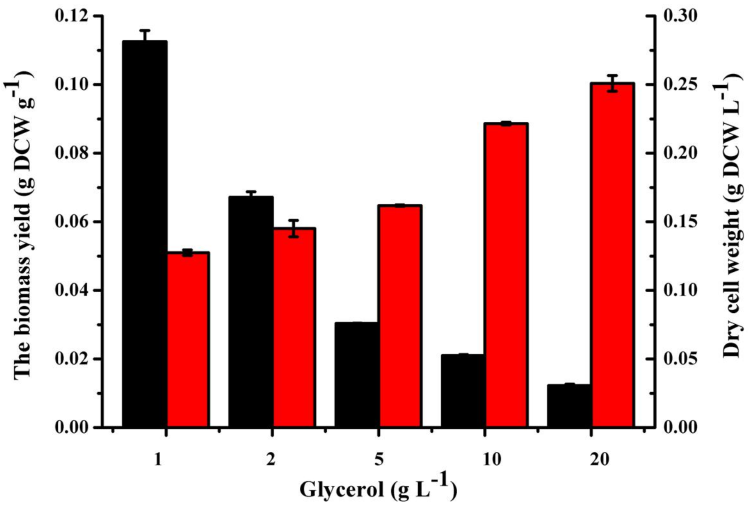

Additional file 5: Fig. S4 The dry cell weight and biomass yield of *G. oxydans* 621H in mineral salts medium containing different concentrations of glycerol. Dry cell weight (red), biomass yield (black). The biomass yield was calculated based on concentrations of the cells and added glycerol. Calculation of dry cell weight: g DCW $L^{-1} = 0.3896 \times \Delta OD_{600nm} - 0.0004$.
